# Pharmacoepidemiology resources in Ireland—an introduction to pharmacy claims data

**DOI:** 10.1007/s00228-017-2310-7

**Published:** 2017-08-17

**Authors:** Sarah-Jo Sinnott, Kathleen Bennett, Caitriona Cahir

**Affiliations:** 1 Department of Non-communicable Disease Epidemiology, London School of Hygiene and Tropical Medicine, Keppel St, London, WC1E 7HT UK; 2 Division of Population Health Sciences, Royal College of Surgeons Ireland, Dublin, Ireland

**Keywords:** Database, Pharmacoepidemiology, Drug utilisation, Claims data

## Abstract

**Introduction:**

Administrative health data, such as pharmacy claims data, present a valuable resource for conducting pharmacoepidemiological and health services research. Often, data are available for whole populations allowing population level analyses. Moreover, their routine collection ensures that the data reflect health care utilisation in the real-world setting compared to data collected in clinical trials.

**Setting and methods:**

The Irish Health Service Executive-Primary Care Reimbursement Service (HSE-PCRS) community pharmacy claims database is described. The availability of demographic variables and drug-related information is discussed. The strengths and limitations associated using this database for conducting research are presented, in particular, internal and external validity. Examples of recently conducted research using the HSE-PCRS pharmacy claims database are used to illustrate the breadth of its use.

**Results and conclusions:**

The HSE-PCRS national pharmacy claims database is a large, high-quality, valid and accurate data source for measuring drug exposure in specific populations in Ireland. The main limitation is the lack of generalisability for those aged <70 years and the lack of information on indication or outcome.

## Introduction

Pharmacy claims data offer substantial potential for studying the effects of drugs in large populations, and in specific populations such as the elderly which are often not represented in clinical trials [1]. The Health Service Executive-Primary Care Reimbursement Service (HSE-PCRS) pharmacy claims database in Ireland is widely used in pharmacoepidemiology research, drug utilisation research and health services research.

The aims of this review are to (i) provide an overview of the health system in Ireland, setting the context for the use of the national HSE-PCRS pharmacy claims database in health care provision; (ii) describe the HSE-PCRS database and (iii) describe the validity of the database, including both internal and external validity. Lastly, examples of research conducted using the database are provided.

## Description of the health system and reimbursement of medicines

Ireland is the only European country that does not provide universal access to primary health care [2]. Instead, the Irish healthcare system, which is predominantly tax funded, operates on a complex two-tiered basis [3]. Across these public and private tiers, the majority of pharmaceutical expenditure is funded through the National Shared Services HSE-PCRS. Three principal community drug schemes exist under this system: (i) General Medical Services (GMS) scheme, (ii) Drug Payment (DP) scheme and (iii) Long-Term Illness (LTI) scheme. Altogether, the community drug schemes account for approximately 82% of total public expenditure on the acquisition cost of pharmaceuticals prescribed by GPs [4]. Other smaller specific schemes also exist, for example the methadone treatment scheme for opioid dependence and a high-technology drugs scheme which provides access to high-cost innovative treatments, e.g. immunologics and cancer treatments, but these not discussed further here [4].

### General Medical Services scheme

In 2015, almost 40% of the population (1.7 million people) were in receipt of public health insurance, or covered by the GMS scheme (known as medical card holders) [5]. The GMS scheme entitles the individual to primary care services and hospital services free at the point of access. The exception to this is that prescription medications are subject to a flat copayment. The current copayment (as of March 2017) is €2.50 per item (capped at €25 per month per household) and €2.00 per item for those aged over 70 years (capped at €20 per month). Copayment exemptions are extended to children in the care of the HSE, for example in foster care, and people living in emergency reception accommodation under the Irish Refugee Protection Programme. The supply of methadone for opioid dependence is also not subject to copayment. Qualification for the GMS scheme is on the basis of income-related means-testing [4]. Automatic entitlement for those aged ≥70 years occurred between July 2001 and December 2008; however, since January 2009, means-testing was introduced for those aged ≥70 years but with a higher income threshold than those aged ≥66 years and those aged <66 years. Discretionary medical cards may be awarded in certain cases, for example at diagnosis of cancer [4].

### Drug Payment scheme

The remainder of the population not covered by the GMS scheme are referred to as being in the “private” tier. Although hospital services are free to all individuals in Ireland, a proportion (approximately 46% in 2015) choose to purchase private health insurance; the main advantage of which is quicker access to secondary and tertiary specialist services [3, 6]. For the most part, private health insurance does not cover primary care services; thus, out-of-pocket payments for general practitioner (GP) visits are standard practice. Individuals in the private health care system can receive government-subsidised access to prescription medications through the DP scheme. Under the DP scheme, an individual or a family is responsible for paying up to €144 per month for their medications. Once this limit is reached, the individual or family incurs no further cost for the duration of the month [4]. In 2014, 29% of the population accessed medication under this scheme [4].

### Long-Term Illness scheme

The LTI scheme is awarded, independently of income, to individuals with one of 16 chronic illnesses, for example diabetes mellitus or epilepsy. Individuals in receipt of care on this scheme, approximately 3% of the population, are provided with prescription medicines without any copayment. However, individuals are responsible for GP consultation fees [4].

## Description of the database

Community pharmacists dispensing medications on the main drug schemes submit their claims every month to the HSE-PCRS for reimbursement. The vast majority do so electronically. The HSE-PCRS database thus holds data on every prescription filled in Ireland in the community on the GMS scheme and the LTI scheme. However, data for medications dispensed on the DP scheme are incomplete because medications are paid for in full by the individual/family up to the monthly threshold. Hence, there is no requirement to submit a claim to the HSE-PCRS for medications already paid for by the individual. Drugs administered in hospital are not available in this database. However, it is possible to explore in-hospital-initiated therapies in GMS patients only by examining claims on the “hospital emergency scheme”. This is a further community drug scheme available to GMS patients that provides 7 days’ supply of medication on foot of a hospital prescription, before the prescription must be written by the GP.

### Drug data

For each drug dispensed in Ireland and reimbursed by HSE-PCRS, data are available in the database for the date of dispensing, quantity of medication provided, strength, dosage form, route of administration, ingredient cost, community drug scheme on which drug was dispensed and dispensing fees to the pharmacist. Prescriptions are coded using the World Health Organisation Anatomical Therapeutic Chemical (WHO-ATC) classification system and corresponding defined daily dose (DDD) [7]. Data on the dispensing pharmacy and the prescriber are also collected. Missing data are negligible for all medication-related fields.

### Demographic data

Information on age and gender at the individual level are available in the database, with negligible missingness. Information on the HSE region and local health office for each patient is also available. Unfortunately, clinical data and data on lifestyle behaviours are not available in this database.

### Validation studies

#### Internal validity

Pharmacy claims databases are thought to be highly accurate because the data collected must be complete, correct and up-to-date for reimbursement purposes [1]. In a study of 97 patients in two Irish hospitals, it was found that the HSE-PCRS database was the most accurate method of medicine reconciliation relative to GP records and records from individual community pharmacies [8]. Additionally, the HSE-PCRS database has been found to be more accurate and complete than patient self-report, perhaps due to issues with patient recall, in a study of 2641 patients aged over 50 years. [8, 9].

Furthermore, prior research indicates an association between drug exposure measured through dispensed drug records and clinical outcomes [10, 11]. Thus, pharmacoepidemiological studies using pharmacy claims data can reasonably attempt to establish associations between causes (medications) and outcomes (side effects or medication effectiveness). One limitation of HSE-PCRS data for assessing exposure to medications is worth noting. At present, there is no number of days’ supply or duration of use variable, which is common in international prescription claims databases [12]. The date on which a medication is dispensed along with the quantity provided can be used to calculate duration. In addition, the number of Daily Defined Doses (DDDs) can be calculated as a proxy for days’ supply by multiplying the strength of the medication dispensed by the quantity dispensed and then dividing this number by the DDD. For example, if a person is dispensed 112 tablets of 500 mg metformin, which has a DDD of 2000 mg, the person received 28 DDDs [i.e. (112 × 500) / 2000]. This method has been demonstrated to have mixed accuracy for several different medication types [12].

Other limitations are common to all international databases of prescription claims [1]. For example, information on medications not reimbursable by the HSE-PCRS is not available in the database. This is somewhat ameliorated in the Irish database due to a comprehensive reimbursable medications list. The relatively low copayment also means that patients on the GMS scheme will sometimes opt to get a prescription for an item that can be bought over the counter, if the copayment is less than the item cost and eligible for reimbursement. There is no information on the indication for the medication or on outcomes of any medications.

#### External validity

The GMS population is approximately 40% of the population [4], but those who are socio-economically disadvantaged and those aged ≥70 years are over-represented; hence, there may be some limitations as regards generalisability in pharmacoepidemiological studies [13]. For those aged ≥70 years, the database is representative of the Irish population at the same age, attributable to the fact that almost every person aged ≥70 years is eligible for the GMS scheme [14]. As of 2013, 90% of men and 94% of women aged ≥70 years were eligible [14]. Therefore, studies in those aged ≥70 years have greater external validity than for other age groups. For example, a study comparing the prevalence of potentially inappropriate prescribing in the Republic of Ireland and Northern Ireland (which is part of the UK) found that potentially inappropriate prescribing rates were similar between the two populations, when restricted to those aged ≥70 years [15].

However, less than 50% of the Irish population aged <70 years of age are covered by the GMS scheme (Table 1). The impact of this limitation on generalisability was highlighted in a study comparing potentially inappropriate prescribing between the Republic of Ireland and Northern Ireland; differences in the prevalence of potentially inappropriate prescribing between the settings were attributed to a larger proportion of socially disadvantaged middle-aged adults in the Irish database [16]. Additionally, greater proportions of those covered by the GMS scheme are smokers and female than in the general population [13]. In studies of drug effects, care must be taken to ensure no prior evidence of confounding or effect modification by smoking status. If no confounding or effect modification exists (In studies of drug effects, care must be taken to ensure no prior evidence of confounding or effect modification by lifestyle factors such as smoking.), one could reasonably assume that the effects of a drug in this population will be no different to a general population [13].

**Table 1:** Proportion of Irish men and women on GMS scheme

Age group (years)	% Men	% Women
0–15	40.4	39.8
16–24	36.9	43.2
25–34	27.4	32.9
35–44	30.1	33.6
45–54	31.0	31.4
55–64	33.6	35.7
65–69	43.6	55.8
70+	89.9	93.8
Total	38.3	42.2

Source: Health Service Executive and Central Statistics Office, available at http://www.cso.ie/en/releasesandpublications/ep/p-wamii/womenandmeninireland2013/healthlist/health/#d.en.65619

The % column represents the % men/women in the Irish population on the General Medical Services (GMS) scheme

*GMS* General Medical Services scheme

## Examples of research using the HSE-PCRS database

### Chronic disease epidemiology

Methods to calculate burden of disease metrics frequently require weighting of the data to reflect underlying population trends that cannot be detected in sample-based surveys or cohort studies. The HSE-PCRS database allows calculation (rather than estimation) of disease prevalence in specific populations such as those aged ≥70 years and those with specific diseases. For example, Naughton et al. calculated the prevalence of chronic disease in those aged >70 years [17]. Sinnott et al. calculated the prevalence and incidence of type 2 diabetes mellitus using GMS and LTI data, which when combined, provide data on every diabetes medication dispensed in Ireland, thus capturing all treated individuals with diabetes [18]. A limitation of the data in this respect is that without codes for diagnosis/indication, some misclassification is possible, for example women with polycystic ovary syndrome who are treated with metformin may be included in the diabetes numerator [18].

### Health services research

Health services research in Ireland has developed rapidly in recent years. The availability of the HSE-PCRS database has enabled the conduct of high-quality drug utilisation research, research into adherence to medicines and also policy analysis.

#### Drug utilisation research

Drug utilisation research refers to the study of the use of medicines in populations, with special emphasis on the resulting medical, social and economic consequences [19]. Trends in the prescribing of (1) psychotropic medicines and antibiotics in paediatric populations, (2) oral contraceptive use and (3) the prevalence of polypharmacy (≥5 concurrent prescribed medications) have all been recently explored using HSE-PCRS data [20–23]. The effectiveness of randomised interventions designed to improve the prescribing of (i) antibiotics and (ii) preventive therapies in cardiovascular disease was evaluated using data held in the HSE-PCRS database for each GP practice involved in each of the trials [24, 25]. Such trials are good examples of how interventional studies in the area of pharmacoepidemiology/drug utilisation research can be carried out pragmatically, contingent upon the availability of an up-to-date claims database and consent of involved parties.

The Health Products Regulatory Authority, the regulatory agency for medicines and medical devices in Ireland, frequently publishes drug safety warnings [26]. How these warnings affect the prescribing and dispensing of medicines was analysed using the HSE-PCRS database in 2011, with a summary finding that inconsistent effects on the use of various drugs after safety warnings occurs [27]. In contrast, publicity surrounding clinical trials of hormone replacement therapy (HRT) in menopausal women was found to have a significant negative effect on the prescribing of HRT in this patient group [28].

The availability of the HSE-PCRS database has also facilitated research into potentially inappropriate prescribing, and associated costs [29–31]. Relying on the national nature of the data, and regional information within the database, Cahir et al. used the HSE-PCRS database to explore regional variation in potentially inappropriate prescribing to adults ≥70 years [32]. This study found up to fourfold differences in rates of potentially inappropriate prescribing, but this was mostly insignificant after adjusting for patient level variables [32].

The practical value of the database in making predictions for future costs to government has recently been explored. The demographic structure of Irish society is rapidly changing, owing mostly to an ageing population [33]. The impact of this demographic change on medication costs on the GMS scheme has been forecast using current HSE-PCRS data and Central Statistics Office population projections. It is estimated that the GMS medications bill will rise from €1.3 billion in 2016 to €1.9 billion in 2026 [34].

#### Adherence and medication-taking behaviour

Pharmacy claims data offer a useful tool for assessing medication-taking behaviour in patients with chronic diseases and are particularly useful for the evaluation of drugs intended for long-term therapy. Medication-taking behaviour can be defined in terms of two distinct variables: (1) adherence which is acting in accordance with the agreed prescribed interval and dosage of the treatment and (2) persistence which is continuing the treatment for the prescribed duration of time [35]. The HSE-PCRS has been used successfully to describe adherence and persistence to medication, treatment initiation, treatment switching, socio-demographic predictors and regional variation in medication-taking behaviour in various chronic diseases and cancers over time [36–40]. It is important to acknowledge, however, that adherence and persistence measures based on dispensed drug data reflect drug availability as opposed to true exposure.

#### Pharmaceutical policy analysis

After the global economic recession in 2008, several policy measures were implemented in Ireland to help reduce expenditure on publicly reimbursed medications. First, a copayment on the GMS scheme was introduced in October 2010 at 50c per prescription item. Using HSE-PCRS data to evaluate the impact of this policy, Sinnott et al. found that adherence to less-essential medicines was affected to a greater degree than adherence to essential medicines. The exception to this pattern was that adherence to anti-depressant medicines was reduced substantially after the introduction of the copayment, and its subsequent increase to €1.50 [41]. Second, reference pricing and generic substitution were legislated for and introduced on the community drug schemes in 2013. An assessment of cost data in the HSE-PCRS database demonstrated substantial savings of 53% to the exchequer after the introduction of this policy [42]. In absolute terms, this amounted to €35million [42]. Current policies such as the administration of the seasonal influenza vaccine through community pharmacies may be analysable in the future for clinical and cost-effectiveness.

#### Pharmacoeconomics and health technology assessment

Pharmacoeconomic analyses are used to decide whether the use of a new drug is likely to represent value for money for the health care system. Such analyses are generally comparative in nature, requiring information on the costs and effects of alternative treatments in specific indications, which is a typical task of a drug utilisation study [43]. Real-world pharmacy claims data are also helpful in establishing whether evidence from the highly selected populations in phase III randomised controlled trials are transferrable to the unselected populations of everyday clinical practice [44]. The numbers of eligible recipients of new drugs can be calculated from existing pharmacy claims data to provide an estimate of the likely budget impact of introducing or reimbursement of these new medicines [18].

#### Current linkages

The National Cancer Registry Ireland (NCRI), a population-based cancer registry, has been linked with HSE-PCRS data, so that detailed information on medication use is available for those with GMS eligibility and a diagnosis of cancer. This linked database facilitates detailed and high-quality pharmacoepidemiological studies of cancer survival with nuanced drug exposure data available in combination with comprehensive clinical data on type and staging of cancer [45, 46]. A second linkage has been established with The Irish Longitudinal Study of Ageing (TILDA), a nationally representative cohort study of over 8000 adults aged >50 years [47]. This linkage has permitted the conduct of high-quality health services research, in particular of potentially inappropriate prescribing [29, 48]. Linkages have also been established for hospital [49] and mortality data [50].

## Conclusions

The national pharmacy claims database in Ireland is large and highly accurate. Although it is limited by its generalisability for those aged less than 70 years, it is almost completely representative of those aged ≥70 years. This is significant because older people will make up more than 20% of Ireland’s population by 2040 [33]. Furthermore, older people use the most medications in the population [51]. Thus, the ability to carry out pharmacoepidemiological research in this population is of paramount importance. For pharmacoepidemiological research seeking to make associations between drug causes and effects (side effects, effectiveness), the generalisability to the younger population is of concern if is of concern if lifestyle factors such as smoking are confounding variables smoking are confounding variables or effect modifiers of the medication under study.

There are no clinical data in the HSE-PCRS database, but linkages to other databases are possible where patient consent is available. The recent legislation for a unique health identifier in Ireland will assist in the development of a more complete clinical medication database [52]. Moreover, the current move to incorporate an electronic health record for each individual presents a new paradigm for pharmacoepidemiological research in Ireland, within a framework of anonymity, consent and appropriate governance [53]. Such linked databases and electronic health record data present unparalleled opportunities to carry out much needed medication effectiveness studies to study rare outcomes, drug safety and drug effects in large and heterogeneous populations. For example, the number of heart attacks and strokes in the community reduced by blood pressure-lowering therapy [54].

The research carried out to date using the HSE-PCRS database has contributed significantly to national policy making. Global knowledge is amplified through pharmacoepidemiology studies in cancer, health service research in inappropriate prescribing and pharmacoeconomic analyses at the end of life stage [50]. It is now important to encourage policy at the end of life stage and decision makers to facilitate and accommodate future linkage of data. This will benefit the conduct of pharmacoepidemiological research in Ireland, ultimately benefitting not only Irish society, but our understanding of drug effects and safety worldwide.
